# A Retrospective Analysis of Guidewireless Versus Guidewire-Assisted Navigated Percutaneous Pedicle Screw Placement for Minimally Invasive Spine Surgery at a Tertiary Care Institute

**DOI:** 10.7759/cureus.107567

**Published:** 2026-04-23

**Authors:** Mirant B Dave, Mikeson Panthackel, Bharat R Dave, Degulmadi Devanand, Shivanand C Mayi, Ajay Krishnan, Ravi Ranjan Rai, Arjit Vashishtha, Amritesh Singh, Saurabh S Kulkarni, Yogenkumar Adodariya

**Affiliations:** 1 Spine Surgery, Stavya Spine Hospital and Research Institute, Ahmedabad, IND; 2 Spine Surgery, Bhavnagar Institute of Medical Sciences, Bhavnagar, IND; 3 Orthopaedics, Geetanjali Medical College and Hospital, Udaipur, IND; 4 Orthopaedics, University College of Medical Sciences and Guru Teg Bahadur Hospital, New Delhi, IND; 5 Orthopaedics, Mahatma Gandhi Medical College and Research Institute, Aurangabad, IND

**Keywords:** awl tip-tap, guidewireless pedicle screw, guidewire method pedicle screw, navigation, o-arm

## Abstract

Introduction: Pedicle screw fixation is a cornerstone of spinal fusion surgery due to its superior biomechanical stability. While conventional Kirschner wire (K-wire)-guided techniques are widely used, they involve multiple steps and are associated with specific complications. The introduction of the tip-tap awl allows for a guidewireless, single-pass insertion technique, potentially improving surgical efficiency while maintaining accuracy.

Materials and methods: A retrospective analysis was conducted on patients who underwent minimally invasive spine surgery (MISS) with navigated pedicle screw (Medtronic, Littleton, USA) insertion at a single tertiary center. Two navigated techniques were compared: the conventional K-wire-guided method and a guidewireless method using a navigated tip-tap awl (Medtronic, Minneapolis, USA). Data on intraoperative workflow, screw insertion time, complications, and breach rates were collected and analyzed.

Results: A total of 488 screws were evaluated in 122 patients. Screws were placed using either the guidewire method (56 patients) or the guidewireless method (66 patients). Both groups showed high accuracy, with minimal breach rates and no clinically significant postoperative complications. The guidewireless method required fewer procedural steps and demonstrated an average reduction in screw insertion time of 1.30 minutes per screw.

Discussion: The tip-tap awl simplified screw insertion by combining entry and tapping into a single navigated step. This streamlining of the procedure contributed to a more efficient surgical workflow. Despite fewer steps, complication and breach rates remained comparable to the traditional technique, indicating that safety and accuracy were not compromised.

Conclusion: In this retrospective cohort, the guidewireless technique was associated with reduced screw insertion times and a favorable safety profile, suggesting it may serve as an efficient alternative to traditional methods.

## Introduction

Pedicle screws represent the standard of care in adult spinal fusion due to their superior biomechanical pullout strength, enabling satisfactory fixation and effective corrective force application [1]. Traditional open techniques involve extensive soft tissue and muscle dissection, leading to paraspinal muscle atrophy, increased blood loss, and prolonged hospital stay. To avoid these complications, minimally invasive spine surgery (MISS) techniques have been developed [2].

Since its initial description over a decade ago, the technique of MISS screw insertion has undergone substantial evolution. The initial technique required the use of fluoroscopy to place a Kirschner wire (K-wire) with the help of a Jamshedji needle, followed by screw insertion over it [3]. Multiple complications have been associated with the use of this technique, including K-wire fracture, spinal hematomas, neurological injuries, dural injury resulting in CSF leak, and anterior vertebral body breach [4]. Advancements in intraoperative 3D imaging and navigation systems have facilitated the development of improved percutaneous screw techniques.

Studies on the error rates of screw insertion using 2D techniques versus 3D techniques have shown significant advantages with 3D techniques (93.9% vs. 96.4%) [5]. Another systematic review of 68 studies also reached similar conclusions, with reported accuracy rates of 91.4% for fluoroscopy versus 97.3% with CT navigation [6].

Multiple techniques for the insertion of navigated pedicle screws utilizing various navigated instruments are available, and their use is often based on individual surgeon preference and familiarity. Sharp awl, cannulated tap, pedicle access kit (PAK) needle, and tip-tap awl are commonly used in various combinations.

In the present study, we analyzed the use of a novel instrument, the navigated tip-tap awl (Medtronic, Minneapolis, USA), in percutaneous pedicle screw (Medtronic, Littleton, USA) insertion. Consisting of a tap with a sharp tip that facilitates simultaneous pedicle entry and tapping, the tip-tap awl eliminates the K-wire insertion step and the need for separate tapping seen in traditional procedures. This method may reduce complication rates and operative time.

This study aimed to compare operative efficiency and accuracy between guidewire-assisted and guidewireless navigated pedicle screw placement in single-level MISS transforaminal lumbar interbody fusion (TLIF).

## Materials and methods

In this study, we retrospectively reviewed the electronic medical records of patients who underwent K-wireless or K-wire-guided pedicle screw fixation from June 2024 to October 2025 at Stavya Spine Hospital and Research Institute, Ahmedabad, India. The study included patients who underwent single-level MISS TLIF. Patients were excluded if they required multilevel fixation or underwent interbody fusion utilizing only bone grafting. Furthermore, cases involving dysplastic spondylolisthesis, sclerotic pedicles (e.g., secondary to infection), or revision surgeries with previously instrumented pedicles or attempted fusions at the index level were excluded to eliminate anatomical confounders. 

Preoperative demographic data collected included patient age, sex, and BMI. The primary outcome evaluated in this study was the mean screw insertion time. Secondary outcomes included screw placement accuracy (evaluated for breaches on postoperative O-arm® CT scans (O-arm® Surgical Imaging System; Medtronic, Littleton, USA)), total surgery duration, estimated blood loss, and the incidence of intraoperative complications. All procedures were performed by a single senior consultant spine surgeon at our institution. The surgeon had surpassed the initial learning curve for navigated MISS procedures prior to the commencement of this study. Postoperative CT scans were analyzed for screw breaches using the Gertzbein-Robbins system (GRS) [7] by a group of five surgeons. The grading was performed collectively by the panel of five spine surgeons, and a final grade was assigned by strict consensus. The primary outcome, average screw insertion time, was calculated by measuring the total time elapsed from the initial skin incision for the first screw to the final seating of the last pedicle screw and removal of the driver, and subsequently dividing this total duration by the number of screws placed in that case. Intraoperative blood loss from the screw insertion sites was estimated by visually quantifying surgical gauze usage. Intraoperative blood loss was estimated using visual quantification of standard 4x4-inch surgical gauze, with each fully saturated piece estimated to hold 10 mL of blood. The two techniques were performed concurrently throughout the entire study period (June 2024 to October 2025), thereby minimizing temporal bias. As previously stated, technique allocation was strictly dictated by the daily availability of the respective instrumentation sets from the central sterilization department, independent of patient pathology or anatomy.

Surgical technique

All cases were performed using the O-arm® Surgical Imaging System integrated with the StealthStation® Navigation System (Medtronic Navigation, Louisville, USA) and standard Medtronic spinal instrumentation. All pedicle screws were inserted following the placement of the interbody graft. The navigation frame was fixed on the spinous process above the level of instrumentation. The O-arm® was brought into the surgical field, an intraoperative 3D CT scan was performed, and the images were transferred to the StealthStation® Navigation System for guidance.

Guidewireless Method

**Figure 1:**
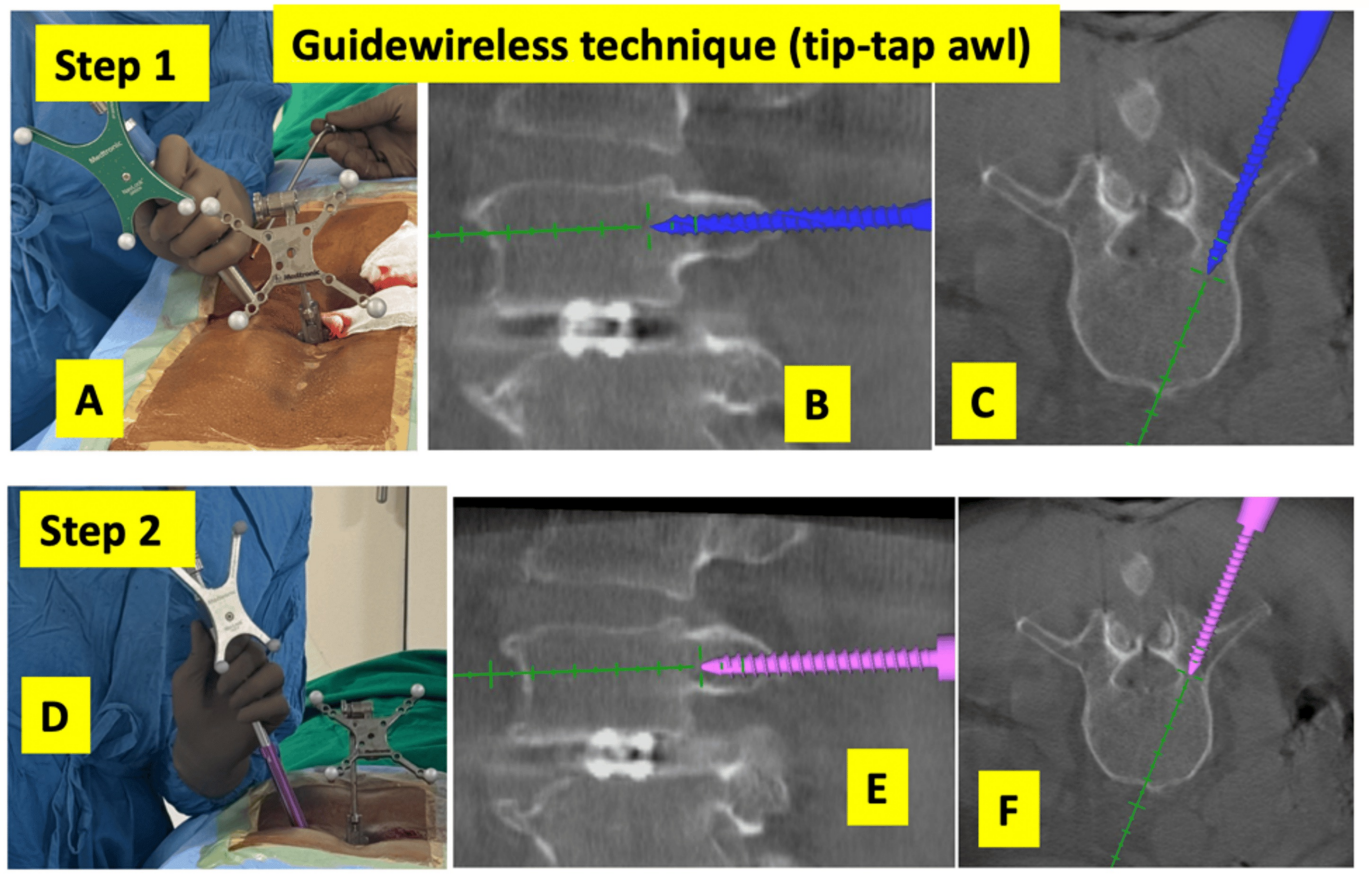
Guidewireless navigated pedicle screw insertion technique. Step 1: A navigated awl tip-tap instrument is used to simultaneously establish the pedicle entry point and prepare the screw trajectory in a single step. (A) Intraoperative image showing the use of the tip-tap awl with a sleeve. Simultaneous navigation display of sagittal (B) and axial (C) images showing the navigated tip-tap awl advancing along the planned screw trajectory. Step 2: A navigated pedicle screw is inserted along the prepared trajectory. (D) Intraoperative image demonstrating screw insertion using the navigated driver. Simultaneous navigation display of sagittal (E) and axial (F) images showing the screw being inserted along the tapped trajectory.

Percutaneous skin incisions were made after marking the skin entry point using navigation guidance. Using the navigated tip-tap awl, the entry hole was made and the trajectory tapped simultaneously. Relying on tactile feedback, the pilot holes were re-entered with a navigated screwdriver (Medtronic, Littleton, USA) to insert the screws (a total of two steps). The steps are shown in Figure 1.

Guidewire-Guided Method

**Figure 2:**
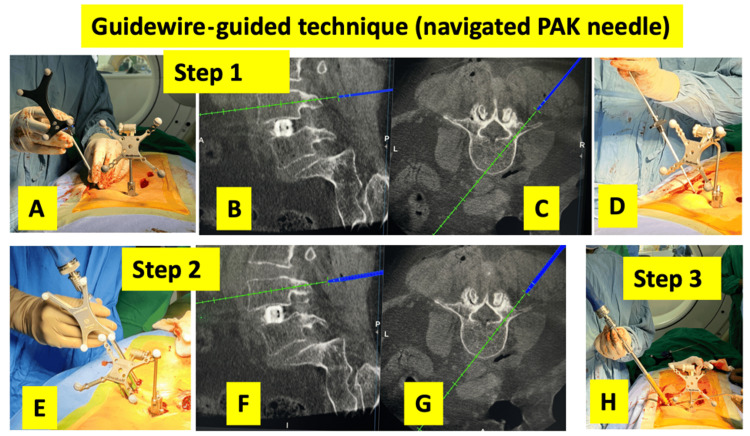
Guidewire-assisted navigated pedicle screw insertion technique. Step 1: A navigated pedicle access kit (PAK) needle is used to establish the pedicle entry point and define the desired screw trajectory, following which a guidewire is advanced along the planned path. (A) Clinical image showing the use of the navigated PAK needle to insert the guidewire. Simultaneous navigation display of sagittal (B) and axial (C) images of the PAK needle. (D) Insertion of the guidewire after the PAK needle is placed in the planned trajectory. Step 2: A cannulated tap is passed over the guidewire to prepare the pedicle tract to the appropriate diameter. (E) Clinical image showing the insertion of the navigated cannulated tap to insert the guidewire. Simultaneous navigation display of sagittal (F) and axial (G) images of the cannulated tap. Step 3: (H) A cannulated pedicle screw is inserted over the guidewire along the prepared trajectory.

A navigated pedicle access kit (PAK) needle (Medtronic, Minneapolis, USA) was used to make pilot holes and pass the guidewires along the desired trajectory in the pedicles. Next, the trajectory was prepared using a navigated tap (preferably 4.5-5.5 mm) (Medtronic, Littleton, USA). Finally, cannulated pedicle screws were passed over the guidewire (a total of three steps). The steps are shown in Figure 2.

Statistical analyses

Statistical analyses were performed using IBM SPSS Statistics version 20.0 (IBM Corp., Armonk, USA). The primary dependent variable (outcome) was defined as the mean per-patient screw insertion time. Continuous variables (such as age and BMI) were assessed for normality using the Shapiro-Wilk test and are presented as means with standard deviations. Categorical variables (such as sex and operative level) are presented as frequencies and percentages. Univariate comparisons between the guidewire-assisted and guidewireless cohorts were performed using independent samples t-tests for normally distributed continuous data, and chi-square or Fisher’s exact tests for categorical data. A p-value of < 0.05 was considered statistically significant.

## Results

A total of 488 screws were analyzed in 122 patients (51 male, 71 female). Of these, 264 screws (66 patients) were inserted using the guidewireless method, and the remaining 224 screws (56 patients) were inserted using the guidewire method. Patient age ranged from 19 to 85 years. In total, two screws were placed in L2, 24 in L3, 182 in L4, 220 in L5, and 60 in S1. The distribution of operated levels was as follows: L2-L3 (1), L3-L4 (11), L4-L5 (80), and L5-S1 (30). A total of four screws were noted to have grade 1 breaches (three in guidewire-guided surgeries and one in guidewireless surgery). All breaches were on the lateral cortex. The remaining 484 screws had no breaches. None of the breaches resulted in any clinically significant complaints in the postoperative period. The total breach rate was 0.4% (1/264) in the guidewireless group and 1.2% (3/224) in the guidewire group; however, this difference was not statistically significant (p = 0.33, Fisher's exact test). The number of screws placed in each surgery was four. Assumption testing using the Shapiro-Wilk test confirmed that continuous variables were normally distributed. The average time per screw was 5.68 minutes for guidewire surgery and 4.38 minutes for guidewireless surgery. Demographic data are shown in Table 1.

**Table 1:** Baseline demographics and operative details. Continuous data are presented as mean ± standard deviation. Categorical data are presented as frequency (percentage). Univariate comparisons were performed using the independent samples t-test for continuous variables and the chi-square test for categorical variables. Statistical significance was defined as p < 0.05.

Variable	Guidewire-Assisted Cohort (n = 56)	Guidewireless Cohort (n = 66)	p-value
Age (years)	58.5 ± 8.1	58.7 ± 8.3	0.89
Sex	-	-	0.88
Male	23 (41.1%)	28 (42.4%)	-
Female	33 (58.9%)	38 (57.6%)	-
BMI (kg/m²)	27.8 ± 4.0	27.9 ± 4.1	0.89
Operative level	-	-	0.98
L2-L3	0 (0.0%)	1 (1.5%)	-
L3-L4	5 (8.9%)	6 (9.1%)	-
L4-L5	37 (66.1%)	43 (65.2%)	-
L5-S1	14 (25.0%)	16 (24.2%)	-

K-wire-related complications were noted in only one patient who had K-wire breakage, for which the wire was left in situ.

An independent t-test assuming unequal variances was conducted to compare the mean screw insertion times between the two techniques. The test yielded a t-statistic of 4.73 and a p-value of 0.0000083, indicating a statistically significant difference in screw insertion times between the two methods. The results are shown in Table 2.

**Table 2:** Comparison of the mean of average screw times between the guidewire-assisted and guidewireless navigated techniques.

Metric	Guidewire Method	Guidewireless Method
Mean of the average screw times (min)	5.68	4.38
Standard deviation (min)	1.78	1.12
Number of surgeries	56	66

To evaluate the independent effect of patient anatomy on surgical duration, regardless of the surgical technique utilized, the two groups were combined into a pooled cohort (n = 122) for exploratory analysis. An exploratory comparison within the whole cohort revealed that mean screw insertion time did not differ significantly between BMI categories on Welch’s independent t-test (t = 1.705, df = 69.37, p = 0.0927). The results are shown in Table 3.

**Table 3:** Comparison of the mean screw insertion times based on BMI categories across the pooled study cohort. This exploratory analysis evaluates the independent impact of BMI on overall insertion time across all patients, irrespective of the surgical technique (guidewire or guidewireless) utilized. The values are in minutes per screw.

BMI	Number of Patients (Total = 122)	Average Time for Screws
Less than 30 kg/m²	83	5.14 ± 1.2
More than 30 kg/m²	39	4.72 ± 1.3

An exploratory comparison between the operated levels within the whole cohort also did not yield significant differences. A one-way analysis of variance was performed to compare mean screw insertion times across operated levels (L3-L4, L4-L5, and L5-S1). No statistically significant difference in screw insertion times was observed between levels (p = 0.552). The L2-L3 level was excluded from inferential analysis due to a single observation. The average times for different levels were compared and are shown in Table 4.

**Table 4:** Comparison of the mean screw insertion times based on operative level across the pooled study cohort. This exploratory analysis evaluates the independent impact of the specific operative level on overall insertion time across the pooled cohort, regardless of the surgical technique utilized. The L2-L3 level was excluded from inferential statistical comparison due to insufficient sample size (n = 1). The values of average time are in minutes.

Level	Number of Cases (Total = 122)	Mean of Average Screw Insertion Times
L5-S1	30	4.8 ± 1.3
L4-L5	80	5 ± 1.4
L3-L4	11	4.58 ± 1.2
L2-L3	1	4.7 ± 1.5

The median number of O-arm® spins taken was 2.0, ranging from one to three spins. In all cases, blood loss from the screw site for both techniques was negligible (< 50 mL).

## Discussion

With the advent of navigation for percutaneous pedicle screws, the advantages of 3D navigation in comparison to 2D have been well documented, including increased accuracy rates and lower complication rates. Also, 3D navigation provides the surgical team with the added benefit of minimal radiation exposure, as 2D percutaneous screw placement relies on multiple fluoroscopic shots to confirm trajectory, during which the surgeon and the team are exposed to radiation [8,9]. Multiple techniques for pedicle screw insertion are available, which include K-wire-guided and K-wireless techniques, each with its own set of advantages and disadvantages.

K-wire-related complications, though rare, have been described in the literature, such as K-wire fracture, visceral injury, and vascular injury. The rates of K-wire fracture have been reported to be six in 513 patients [2]. The rate of anterior vertebral body breaches is also very low, with studies noting seven breaches in 525 screws [10]. Regarding hardware complications in our study, we experienced a K-wire breakage rate of only one in 224 screws.

Owing to the reduced number of steps, the guidewireless technique has a theoretical advantage of reducing surgical time. The results of our study show a statistically significant reduction in screw insertion time of 1.30 minutes per screw (average time taken was 5.68 minutes for the guidewire technique and 4.38 minutes for the guidewireless technique). Other studies evaluating procedure times for fluoroscopy-guided K-wireless screws, a technique distinct from our navigation-assisted approach, have reported an average insertion time of 6.92 minutes per screw [11].

In our study, both methods demonstrated high accuracy with minimal breach rates (0.4% in the guidewireless technique versus 1.2% in the guidewire-guided technique; p = 0.33). While these findings suggest a favorable safety profile for the guidewireless technique, the overall incidence of breach events was too low to draw definitive statistical conclusions regarding comparable safety. These results align with existing literature; a study comparing K-wireless and K-wire-guided techniques reported varying breach rates (e.g., 2-12% vs. 3.9%) but found no statistically significant differences between the two methods [12]. Other studies on the accuracy of the tip-tap awl have shown breach rates of about 9% and an accuracy of 98% [13,14]. Screw morphometric analysis has led to a consensus establishing a 2 mm safe zone for medial breaches due to the presence of the epidural space; therefore, minor medial breaches can sometimes be considered acceptable [15,16]. In our study, the breaches noted intraoperatively were isolated to the lateral cortex; hence, none of them were revised.

Similarly, other novel techniques for screw insertion are currently under investigation. One such technique includes a one-step insertion of a self-drilling and self-tapping navigated screw. This system incorporates an inbuilt K-wire over which the screw is advanced, thereby saving time. However, in another study, the authors analyzed four pilot cases and found the average time to be 8.2 minutes per screw [17].

The uniqueness of our study lies in the selection of 55 comparable cases of single-level MISS TLIFs for comparison using both techniques. Although a few studies have been conducted on the tip-tap awl, they have evaluated it only in isolation and not in comparison to other techniques. Hence, the analysis from our study regarding time efficiency is a useful finding.

Several limitations must be acknowledged. First, the retrospective, single-center, non-randomized design introduces potential selection bias, as technique allocation was dictated by daily instrument availability rather than randomization. Second, the extremely low incidence of complications renders the study underpowered to definitively establish statistical non-inferiority regarding safety. Third, the exploratory analyses assessing the impact of BMI and operative level were secondary and not adequately powered, warranting cautious interpretation. Finally, our institution’s specialization in MISS may limit the generalizability of these results. Future prospective randomized controlled trials are required to eliminate confounding variables and validate these findings.

## Conclusions

In conclusion, observational data from this retrospective cohort suggest the guidewireless tip-tap awl technique offers a highly time-efficient alternative for navigated pedicle screw insertion. By reducing procedural steps, it streamlines the MISS workflow without apparent compromise in accuracy. However, while complication and breach rates were minimal, this cohort is underpowered to definitively establish safety non-inferiority. Future prospective randomized controlled trials are necessary to confirm these findings and draw definitive causal comparisons.
